# Neoadjuvant chemotherapy is associated with suppression of the B cell-centered immune landscape in pancreatic ductal adenocarcinoma

**DOI:** 10.3389/fimmu.2024.1378190

**Published:** 2024-04-02

**Authors:** Luise Rupp, Ina Dietsche, Maximilian Kießler, Ulrich Sommer, Alexander Muckenhuber, Katja Steiger, Casper W. F. van Eijck, Leonard Richter, Rouzanna Istvanffy, Carsten Jäger, Helmut Friess, Casper H. J. van Eijck, Ihsan Ekin Demir, Carmen Mota Reyes, Marc Schmitz

**Affiliations:** ^1^ Institute of Immunology, Faculty of Medicine Carl Gustav Carus, Technical University Dresden, Dresden, Germany; ^2^ Department of Surgery, Klinikum rechts der Isar, Technical University of Munich, Munich, Germany; ^3^ Neural Influences in Cancer (NIC), International Research Consortium, Munich, Germany; ^4^ German Cancer Consortium (DKTK), Partner Site Munich, Munich, Germany; ^5^ Institute of Pathology, University Hospital Carl Gustav Carus, Technical University Dresden, Dresden, Germany; ^6^ Institute of Pathology, School of Medicine and Health, Technical University of Munich, Munich, Germany; ^7^ Department of Surgery, Erasmus University Medical Center, Rotterdam, Netherlands; ^8^ Genetic and Molecular Epidemiology Group, Spanish National Cancer Research Center, Centro de Investigación Biomédica en Red Cáncer (CIBERONC), Madrid, Spain; ^9^ Department of General, Visceral and Transplantation Surgery, Heidelberg University Hospital, Heidelberg, Germany; ^10^ Department of General Surgery, Hepato-Pancreato-Biliary (HPB) Unit, School of Medicine, Acibadem Mehmet Ali Aydinlar University, Istanbul, Türkiye; ^11^ Else Kröner Clinician Scientist Professor for Translational Pancreatic Surgery, Technical University of Munich, Munich, Germany; ^12^ National Center for Tumor Diseases (NCT), Partner Site Dresden, Dresden, Germany; ^13^ German Cancer Consortium (DKTK), Partner Site Dresden, German Cancer Research Center (DKFZ), Heidelberg, Germany

**Keywords:** pancreatic ductal adenocarcinoma, tumor immune contexture, B cells, plasma cells, neoadjuvant chemotherapy, tertiary lymphoid structures

## Abstract

Pancreatic ductal adenocarcinoma (PDAC) is typically diagnosed at advanced stages and associated with early distant metastasis and poor survival. Besides clinical factors, the tumor microenvironment (TME) emerged as a crucial determinant of patient survival and therapy response in many tumors, including PDAC. Thus, the presence of tumor-infiltrating lymphocytes and the formation of tertiary lymphoid structures (TLS) is associated with longer survival in PDAC. Although neoadjuvant therapy (NeoTx) has improved the management of locally advanced tumors, detailed insight into its effect on various TME components is limited. While a remodeling towards a proinflammatory state was reported for PDAC-infiltrating T cells, the effect of NeoTx on B cell subsets, including plasma cells, and TLS formation is widely unclear. We thus investigated the frequency, composition, and spatial distribution of PDAC-infiltrating B cells in primary resected (PR) versus neoadjuvant-treated patients using a novel multiplex immunohistochemistry panel. The NeoTx group displayed significantly lower frequencies of pan B cells, GC B cells, plasmablasts, and plasma cells, accompanied by a reduced abundance of TLS. This finding was supported by bulk RNA-sequencing analysis of an independent fresh frozen tissue cohort, which revealed that major B cell pathways were downregulated in the NeoTx group. We further observed that plasma cells frequently formed aggregates that localized close to TLS and that TLS^+^ patients displayed significantly higher plasma cell frequencies compared to TLS^-^ patients in the PR group. Additionally, high densities of CD20^+^ intratumoral B cells were significantly associated with longer overall survival in the PR group. While CD20^+^ B cells held no prognostic value for NeoTx patients, an increased frequency of proliferating CD20^+^Ki67^+^ B cells emerged as an independent prognostic factor for longer survival in the NeoTx group. These results indicate that NeoTx differentially affects PDAC-infiltrating immune cells and may have detrimental effects on the existing B cell landscape and the formation of TLS. Gaining further insight into the underlying molecular mechanisms is crucial to overcome the intrinsic immunotherapy resistance of PDAC and develop novel strategies to improve the long-term outcome of PDAC patients.

## Introduction

Despite promising advances in other cancer entities, pancreatic ductal adenocarcinoma (PDAC) remains one of the most lethal cancers, with an average 5-year survival of approximately 10% (1). This can be attributed to several factors, including detection during advanced stages, early occurrence of distant metastases, and poor response to standard chemotherapy regimens (2). For the low proportion of patients being eligible, complete surgical resection followed by adjuvant chemotherapy is still the predominant curative option (3). Although neoadjuvant (radio)chemotherapy (NeoTx) provides an additional option for locally advanced and borderline resectable tumors, overall survival (OS) remains low, and recurrences are frequent (4). While novel immunotherapies such as checkpoint inhibitors (CPI) enabled a successful treatment of advanced-stage tumors of, for example, lung and melanoma, PDAC displayed discouragingly low response rates to CPI monotherapy so far (5, 6). Since the discovery of CPIs, a great effort has been made to uncover the underlying mechanisms of action and identify potential biomarkers to efficiently predict response rates and stratify patients accordingly (7). It was found that besides molecular determinants such as the tumor mutational burden, the existing tumor immune contexture plays a pivotal role in determining both response to therapy and prognosis (8, 9). Importantly, PDAC is characterized by a dense, fibrous desmoplastic stroma and an immunosuppressive tumor microenvironment (TME), potentially contributing to the poor response to CPI therapy (10, 11). Over the last decade, efforts to identify biomarkers to efficiently predict therapy response and guide personalized treatment approaches in PDAC have been primarily focused on tumor-infiltrating T cells, whereas studies exploring the B cell compartment are rather limited (12–15). Tumor-associated B cells can present scattered in the pancreatic TME or organized in tertiary lymphoid structures (TLS). TLS constitute specialized cellular hubs of T and B cell activation, which can form in close proximity to tumors, orchestrate effective adaptive antitumor immunity, and are thus associated with improved survival and treatment response in many cancers, including PDAC (16–19). Mature TLS consist of distinct T- and B cell zones, activated dendritic cells (DCs), high endothelial venules, and a germinal center (GC) reaction within the B cell follicle and thus harbor a plethora of B cell subtypes.

Previous studies investigating the B cell composition in PDAC relied solely on the use of the pan B cell marker CD20 or transcriptomic data (20–23). However, not only the frequency but also the quality and spatial organization of the B cell response are crucial (24). The main B cell subtypes that can be found in solid tumors are naïve B cells, memory B cells, GC B cells, plasmablasts, plasma cells, and regulatory B cells (Bregs) (25). Naïve B cells, which were not exposed to antigen yet, are predominantly found in secondary lymphoid organs or peripheral blood and represent only a small proportion of B cells present in tumors. Upon antigen encounter and activation, B cells can migrate into B cell follicles to drive the GC reaction. For T cell-dependent antigens, CD4^+^ follicular T helper cells expressing CD40L and a cytokine cocktail stimulate activated B cells to undergo somatic hypermutation and isotype switching. GC B cells proliferate rapidly and are characterized by the expression of CD20, the GC master regulator Bcl6, and the proliferation marker Ki67 (25). Memory B cells express CD20, CD27, and immunoglobulin (Ig)M and mainly circulate in peripheral blood to initiate a response upon secondary exposure to their respective antigen but can also be found within tumor-associated TLS. A subset of B cells differentiates into plasmablasts, which produce antibodies but can still proliferate and are thus characterized by expression of Ki67 in addition to CD20, CD27, MUM1, and CD38. Further differentiated plasma cells are usually located within the tumor stroma and are negative for CD20 and Ki67 but express, in addition to MUM1 and CD38, the characteristic molecule CD138. Due to the lack of specific cell-surface markers for Bregs, they are usually characterized functionally by the secretion of immunosuppressive cytokines like interleukin 10 and transforming growth factor (TGF)-β (26). Thus, Bregs can drive the generation of regulatory T cells and an M2 polarization of macrophages and are commonly associated with immunosuppression and worse clinical outcomes. Another mechanism by which B cells can contribute to tumor progression is the secretion of vascular endothelial growth factor, which promotes angiogenesis (27). In contrast, plasma cells can enable tumor cell killing by IgG-mediated antibody-dependent cellular cytotoxicity and promote antigen-presentation by DCs (28, 29). However, sustained antibody production can also drive chronic inflammation by forming immune complexes and activating the complement system, which may promote cancer development and progression (30). As professional antigen-presenting cells, B cells also engage in the presentation of antigenic peptides and can facilitate the activation of both CD4^+^ and CD8^+^ T cells, ultimately enhancing antitumor immunity (31). In summary, B cells can be associated with both tumor-promoting or antitumor properties, which may contribute to contradictory results regarding the prognostic value of tumor-infiltrating B cells, describing either a positive, negative, or no effect on survival (32, 33).

NeoTx has become the standard of care for PDAC patients with borderline resectable and locally advanced tumors. Importantly, cytotoxic treatment regimens like chemo- and radiotherapy also affect the composition of the TME (34). By inducing immunogenic cell death, chemotherapeutic agents can enhance the antitumor immune response, enable an increased infiltration by T and B cells, and potentially drive the formation of TLS (25). For example, we reported a higher proportion of CD8^+^ T cells and an increased production of proinflammatory cytokines by PDAC-infiltrating T cells in NeoTx patients (15, 23). On the other hand, DNA-damaging agents also affect immune cells and may thus attenuate their frequency and functional properties (35). To gain novel insights into the effect of NeoTx on the B cell compartment, we compared the frequency, composition, and spatial distribution of PDAC-infiltrating B cells in primary resected (PR) and NeoTx-treated PDAC patients. Therefore, we established a novel multiplex immunohistochemistry (mIHC) panel to identify T and B cells, GC B cells, plasmablasts, and plasma cells. We further uncovered the spatial organization of PDAC-infiltrating B cells in intra- and peritumoral areas, both scattered in the fibrotic stroma and organized within TLS. In addition, we analyzed immune-related transcriptomic changes in PDAC patients treated with NeoTx, with a specific focus on B cell immune responses and signaling pathways.

## Materials and methods

### Patient cohort

Tissue samples for mIHC were obtained from PDAC patients who underwent surgical resection with curative intent at Klinikum rechts der Isar (Munich) between 2008 and 2015. In this study, 30 NeoTx patients were matched according to age, sex, and tumor stage with 28 PR cases. NeoTx was administered to patients with radiographically borderline resectable or locally advanced disease. Clinical characteristics and details of the neoadjuvant treatments are outlined in **Tables 1**, **2**, respectively. All patients were informed and provided written consent. This study was approved by the Ethics Committee of the Technical University of Munich (Munich, Germany; Nr. 549/16s).

**Table 1:** Clinicopathological characteristics of PDAC patients for the 7-color B cell panel.

	PR (n=28)	NeoTx (n=30)
**Sex** Male Female	14 (50%) 14 (50%)	16 (53.3%) 14 (46.7%)
**Age** (year)	63 (47–84)	72 (48–83)
**Tumor size** (mm)	40 (22–80)	33 (2-60)
**T Status** T1-2 T3-4	15 (53.6%) 13 (46.4%)	12 (60%) 18 (40%)
**N Status** N0 N1 N2	5 (17.9%) 13 (46.4%) 10 (35.7%)	15 (50%) 15 (50%)
**M Status** M0 M1	27 (96.4%) 1 (3.6%)	26 (86.7%) 4 (13.3%)
**Grading** G1 G2 G3	1 (3.6%) 13 (46.4%) 14 (50%)	0 (0%) 14 (53.8%) 12 (46.2%)
**Resection Status** R0 R1	12 (42.9%) 16 (57.1%)	11 (42.3%) 15 (57.7%)
**Tumor Localization** Head Body Tail	23 (82.1%) 5 (17.9%) 0 (0%)	23 (76.7%) 5 (16.7%) 2 (6.6%)

For continuous variables, the median is displayed with the range in brackets. For categorical variables, the number of patients with the percentage of all patients in brackets is shown.

**Table 2:** Neoadjuvant therapy regimen of PDAC patients for the 7-color B cell panel.

	CTx only (n=25)	Additional RTx (n=5)
Gemcitabine	11 (44%)	2 (40%)
FOLFIRINOX	10 (40%)	2 (40%)
Others	1 (4%)	1 (20%)
No information	3 (12%)	0 (0%)

Clinical information about the cohorts used for bulk RNA-sequencing (RNAseq) and the manual counting of TLS in H&E-stained sections can be found in **Supplementary Tables 1–3**.

### Whole transcriptome sequencing from bulk fresh frozen PDAC samples

We conducted a bulk RNAseq analysis using 45 patient-derived PDAC fresh frozen samples. Tissue specimens were finely minced and then subjected to RNA extraction using a Trizol reagent. The extracted RNA was further purified utilizing the Qiagen RNeasy Mini Kit, with subsequent assessment of concentration and quality through NanoDrop and Agilent Bioanalyzer, respectively. Following this, first-strand cDNA synthesis was performed, and libraries were prepared using the Illumina TruSeq Stranded mRNA Library Prep Kit (Novogene®). The resulting libraries underwent sequencing on the Illumina platform for comprehensive RNA-quality control and whole transcriptome sequencing analysis.

All statistical analyses were performed using the statistical software R (36). Count data was imported into the DESeq2 workflow to normalize data and analyze differentially expressed genes (37). Gene set enrichment analysis was performed using the *clusterProfiler* package (38).

### Multiplex immunohistochemistry

To detect, identify, and quantify PDAC-infiltrating B cell subsets, formalin-fixed paraffin-embedded (FFPE) tissue sections were stained on a Ventana Discovery Ultra platform (Ventana Medical Systems, Basel, Switzerland) as described previously (39). The tyramide signal amplification-based Opal technology (Akoya Biosciences, Marlborough, MA, USA) enables simultaneous imaging of up to six markers in addition to DAPI. Upon deparaffinization and rehydration of FFPE sections, antigen retrieval was performed using Cell Conditioning Solution (CC) 1 (Ventana Medical Systems, pH 9). The staining is then carried out by sequential rounds of primary antibody binding, horse radish peroxidase-coupled secondary antibody incubation, detection by Opal reagent, and heat-mediated antibody stripping. Individual dilutions, incubation times, temperatures, and antibody suppliers are listed in **Supplementary Table 4**. Finally, slides were counterstained with DAPI, mounted in Fluoromount-G® medium (SouthernBiotech, Birmingham, Alabama, USA), and stored at 4°C until image acquisition.

### Image acquisition

Upon whole slide scanning with the Vectra 3.0 Automated Imaging System (Akoya Biosciences) at 100x magnification, regions of interest were defined in Phenochart software (Akoya Bioscience) based on H&E-stained sections. Then, multispectral images (MSIs) were acquired at 200x magnification, spectrally unmixed using inForm software (Akoya Biosciences) and a previously built spectral library, and exported as multi-channel TIFF files.

### Manual TLS counting in H&E-stained tissue sections

For assessment of TLS numbers in H&E-stained sections, slides were scanned at 400x magnification using a Leica autoscanner. Tumoral and peritumoral regions were defined by a trained pathologist and transferred to the scanned slides. TLS were defined as dense lymphocyte accumulations, and GC status was evaluated by the presence of characteristic centroblast aggregates.

### Image analysis and quantification

Quantification of distinct tissue areas, subsequent cell segmentation, and cell type detection were performed using QuPath software (40). Initially, single MSIs were stitched to create spectrally unmixed whole slide images. Then, intra- and peritumoral regions were defined in a pathologist-assisted manner based on standard H&E-stained sections. To distinguish non-tissue, TLS, stromal, and intraepithelial areas, a pixel classifier was trained on a composite training image comprised of 100 individual MSIs and validated on a set of additional 100 regions of interest (**Supplementary Figure 1**). Following, cell segmentation was performed using the StarDist QuPath extension with adjusted parameters (41). Then, object classifiers were trained for either single markers (CD3, CD20, Ki67, CD38) or complex cell types (CD20^+^Ki67^+^Bcl6^+^CD38^-^ GC B cells, CD20^+^Ki67^+^CD38^+^CD138^-^ plasmablasts, and CD20^-^Ki67^-^CD38^+^CD138^+^ plasma cells) and a composite classifier was created. Finally, the trained pixel classifier, StarDist cell segmentation, and composite object classifier were applied to all 58 stitched whole slide images. For representative images, unmixed multi-channel TIFFs were processed in ImageJ software (42). To enable a larger field of view, separate MSIs were stitched using the grid/collection stitching plugin (43).

For manual counting of TLS in the multiplex-stained cohort, the stitched, unmixed whole-slide images were used. Thus, dense accumulations of CD3^+^ T cells and CD20^+^ B cells with a clearly defined delineation edge from other stromal tissue were identified as TLS. GC^+^ TLS were classified as TLS with a dense accumulation of CD20^+^Ki67^+^Bcl6^+^ B cells.

### Data analysis

Upon image analysis, exported data was analyzed using RStudio and R v4.2.1 (36). Thus, cell numbers, densities, and proportions were calculated for all tissue compartments (combined, TLS, stromal, epithelial), and both analyzed tissue regions (peri- and intratumoral) using packages from the *tidyverse* collection (44). In addition, the *survival* and *survminer* packages were used for univariate and multivariate survival analysis by the Kaplan-Meier method or Cox proportional hazards (ph) models (45–47).

## Results

### Characterization of the PDAC-infiltrating B cell composition

**Figure 1:**
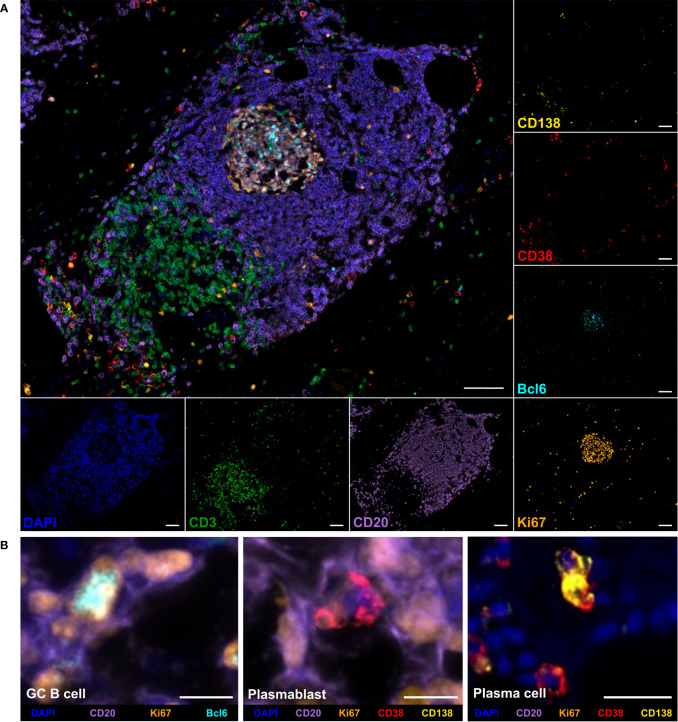
Multiplex immunohistochemistry (mIHC) staining of B cell subpopulations and T cells in a tertiary lymphoid structure (TLS) in pancreatic adenocarcinoma (PDAC) tissue. **(A)** Representative multicolor and single channel images of the B cell panel, including DAPI (blue), CD3 (green), CD20 (purple), Bcl6 (cyan), Ki67 (orange), CD38 (red), and CD138 (yellow). The scale bar is 100 µm. **(B)** Detailed views of germinal center (GC) B cells, plasmablasts, and plasma cells. Scale bars equal 10 µm for GC B cell and plasmablast and 25 µm for plasma cell image.

To analyze the frequency, phenotype, and localization of tumor-infiltrating B cells in PDAC, a novel 7-color mIHC panel including the markers CD3, CD20, Ki67, Bcl6, CD38, and CD138 was established. Besides CD3^+^ T cells and CD20^+^ B cells, GC B cells were characterized as CD20^+^Ki67^+^Bcl6^+^CD38^-^, plasmablasts as CD20^+^Ki67^+^CD38^+^CD138^-^, and plasma cells as CD20^-^Ki67^-^CD38^+^CD138^+^. **Figure 1A** displays a multicolor image and single channels of a TLS and surrounding tissue featuring the major cell types. A detailed view of specific B cell subsets, including the relevant markers, is shown in **Figure 1B**.

To improve the understanding of the spatial organization of the PDAC TME, we analyzed different tissue compartments by training pixel classifiers in QuPath. The epithelial compartment consists of epithelial cells, which may be either non-malignant or malignant. The TLS compartment is defined as dense accumulations of T and B cells with a sharp delineation edge from the stromal area. Thus, the stromal tissue class encompasses all areas that are neither epithelial nor TLS, and the combined class integrates all three tissue compartments. An illustration showing all tissue classes is displayed in **Supplementary Figure 1**. In addition to the microarchitecture of the tumor, we analyzed both intra- and peritumoral regions, which were annotated manually based on H&E-stained sections. The whole tissue region comprises a combination of both areas.

**Figure 2:**
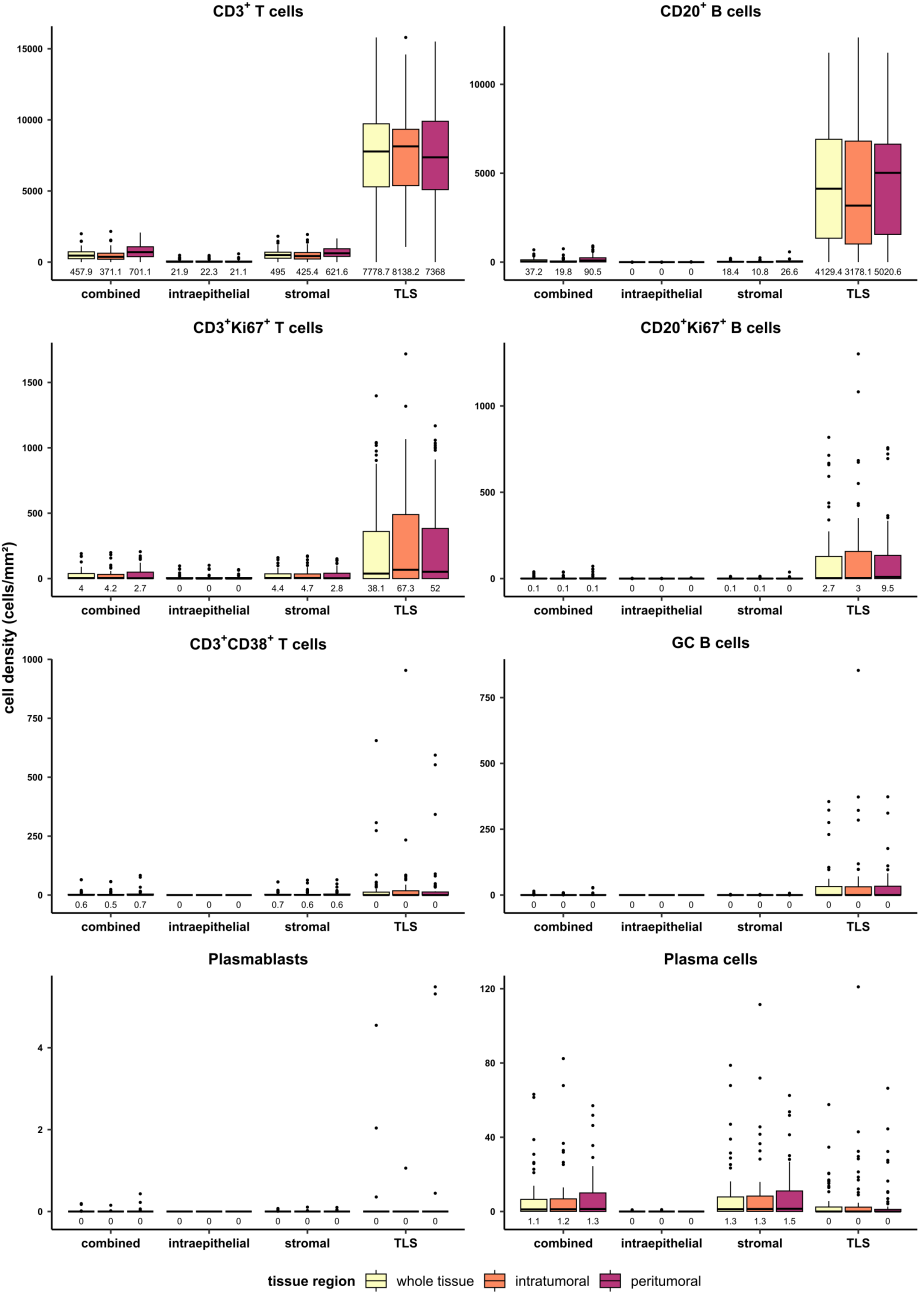
Quantification of T and B cell subtypes in PDAC in different tissue regions (whole tissue, intratumoral, peritumoral) and tissue classes (combined, intraepithelial, stromal, TLS). Cell densities (cells/mm²) were evaluated for T cells, B cells, proliferating T and B cells, CD38^+^ T cells, GC B cells, plasmablasts, and plasma cells in the mixed therapy cohort (n=58). Median values are displayed.

To grasp an overview of the spatial distribution of all analyzed cell types, cell densities (cells/mm²) were compared between different tissue classes and regions in the entire patient cohort (n=58) (**Figure 2**). In the combined compartment of the whole tissue region, PDAC-infiltrating CD3^+^ T cells were generally more abundant than CD20^+^ B cells, with median cell densities of 457.9 cells/mm² and 37.2 cells/mm², respectively. When investigating the spatial distribution, infiltrating lymphocytes were more frequent in the peritumoral region, with CD3^+^ T cells displaying a median cell density of 701.1 cells/mm² in the peritumoral region and 371.1 cells/mm² in the intratumoral area. Similarly, CD20^+^ B cells displayed median densities of 90.5 cells/mm² and 19.8 cells/mm² in the peritumoral and intratumoral regions, respectively, when analyzing the combined compartment which includes TLS. This observation still held true when focusing on non-TLS-associated B cells in the stromal compartment, with median peritumoral CD20^+^ B cell densities of 26.6 cells/mm² and intratumoral densities of 10.8 cells/mm². In general, CD3^+^CD38^+^ T cells, plasmablasts, and GC B cells were extremely rare and only found in few PDAC tissues. Strikingly, the overall infiltration of the epithelial region was low for CD3^+^ T cells and even lower for CD20^+^ B cells. Consequently, this region was not further considered during the subsequent analysis. As expected, the TLS compartment displayed the highest density of both T and B cells in all tissue regions, while plasma cells preferentially resided within stromal areas.

**Figure 3:**
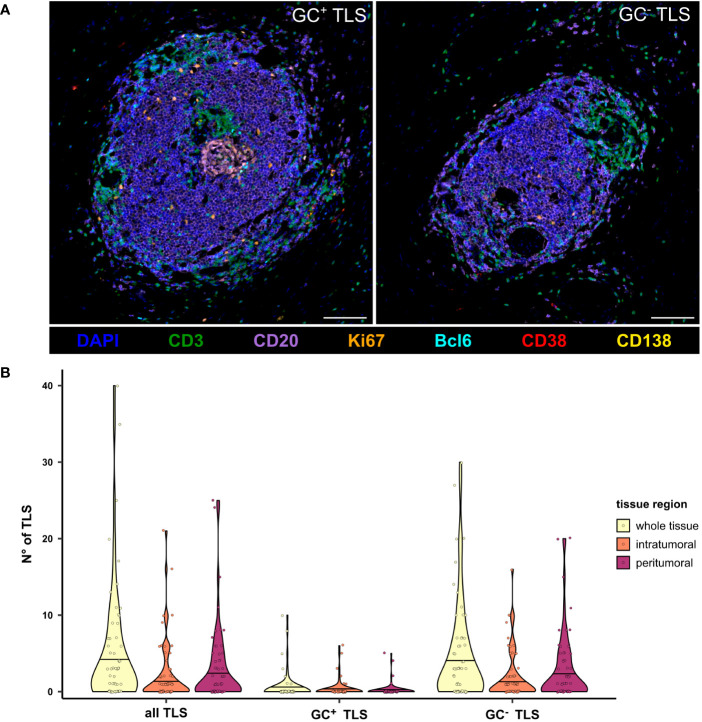
Presence of GC^-^ and GC^+^ TLS in the peritumoral, intratumoral, and whole tissue regions of PDAC tissues. **(A)** Representative multicolor images of a GC^-^ and GC^+^ TLS stained for the B cell panel, including DAPI (blue), CD3 (green), CD20 (purple), Bcl6 (cyan), Ki67 (orange), CD38 (red), and CD138 (yellow). The scale bar is 100 µm. **(B)** TLS were counted manually based on CD3 and CD20 staining, differentiated into GC^+^ and GC^-^ by the presence of Bcl6 and Ki67, and compared between tissue regions in the mixed therapy cohort (n=58).

In addition to the analysis on the single cell level, TLS were counted manually and categorized into GC^+^ and GC^-^ TLS (**Figure 3**). While GC^-^ TLS were present in most of the tissues, GC^+^ TLS were observed only in seven patients (**Figure 3B**).

### Neoadjuvant chemotherapy is associated with a significant reduction of PDAC-infiltrating B cells and TLS frequency

**Figure 4:**
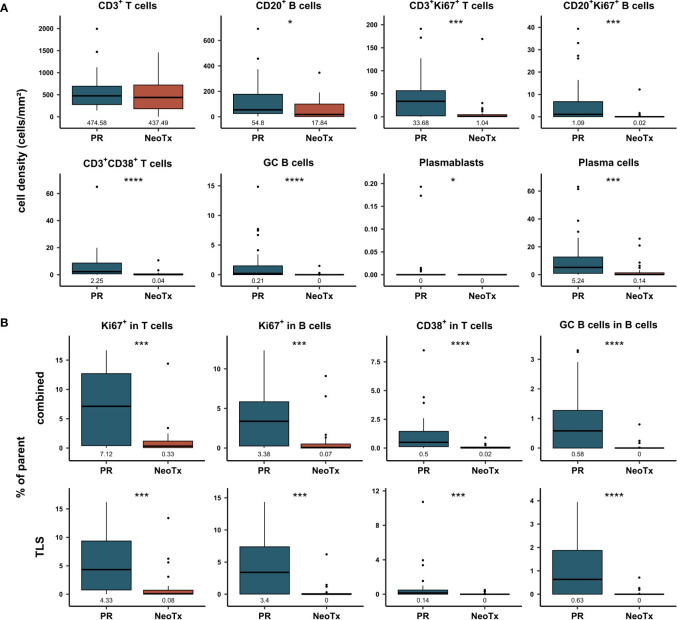
Comparative analysis of T and B cell frequencies and composition in primarily resected (PR) and neoadjuvant-treated (NeoTx) patients. **(A)** Cell densities (cells/mm²) in the combined compartment of the whole tissue region were assessed for the main cell types and compared between the PR (n=28) and NeoTx (n=30) groups. **(B)** Proportion of marker-positive cells is shown in TLS and combined tissue class for the whole tissue region. Median values are displayed, and significant differences were determined using the unpaired Wilcoxon test and are shown as * ≙ p-value ≤ 0.05, ** ≙ p-value ≤ 0.01, *** ≙ p-value ≤ 0.001, and **** ≙ p-value ≤ 0.0001.

Next, we compared the composition of the PDAC-infiltrating lymphocyte landscape between patients who received NeoTx (n=30) and patients who underwent PR (n=28). **Figure 4A** shows the cell density in the combined tissue class and the whole tumor region for both treatment groups. While the overall cell density of CD3^+^ T cells did not differ between both groups, all other analyzed cell types displayed a striking reduction in the NeoTx group. For CD20^+^ B cells, a significant disparity was observed, with median cell densities of 54.8 cells/mm² and 17.84 cells/mm² in the PR and NeoTx groups, respectively. Representative images of a NeoTx patient with low B cell densities and a PR patient with high B cell infiltration are illustrated in **Supplementary Figure 2**. In addition, proliferating lymphocytes displayed highly significant differences, as the median frequency of both CD3^+^Ki67^+^ T cells as well as CD20^+^Ki67^+^ B cells was over 10-fold lower in neoadjuvant-treated patients (**Figure 4A**). Similar results were observed for activated CD3^+^CD38^+^ T cells, GC B cells, plasmablasts, and plasma cells. Importantly, these differences were also present in the stromal region, which excludes TLS (**Supplementary Figure 3A**).

In addition to cell densities, proportions were calculated to assess the functional composition of PDAC-infiltrating lymphocytes. In **Figure 4B**, proportions of the parent population are displayed for the combined and TLS compartments in the whole tissue region. In concordance with cell densities, the proportion of proliferating T and B cells was significantly lower in NeoTx patients, both in the TLS and the combined tissue class (**Figure 4B**). For example, 7.12% of CD3^+^ T cells expressed Ki67 in the PR group, while only 0.33% of CD3^+^ T cells were Ki67^+^ in the NeoTx group. The same trend was observed for activated CD3^+^CD38^+^ T cells and GC B cells. These findings were also detected when excluding TLS, as shown by the separate analysis of the stromal tissue class (**Supplementary Figure 3B**).

**Figure 5:**
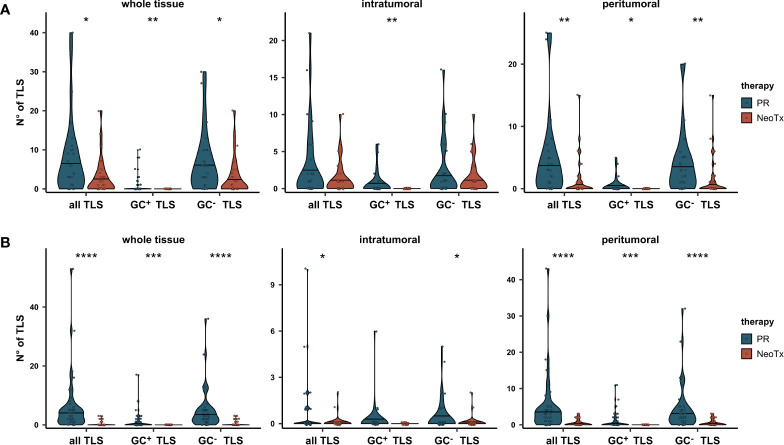
Comparative analysis of TLS abundance between the PR and NeoTx groups. **(A)** TLS were counted manually in the intratumoral, peritumoral, and whole tissue region based on the mIHC staining, differentiated by the presence of a GC, and compared between the PR (n=28) and NeoTx (n=30) groups of our cohort. **(B)** H&E-stained sections of the PREOPANC study were evaluated for TLS presence and compared between treatment arms. Significant differences were determined using the unpaired Wilcoxon test and are shown as * ≙ p-value ≤ 0.05, ** ≙ p-value ≤ 0.01, *** ≙ p-value ≤ 0.001, and **** ≙ p-value ≤ 0.0001.

To assess whether this may be accompanied by a differential abundance of TLS, we compared the number of TLS between the PR and NeoTx groups in all tissue regions (**Figure 5A**). A significant reduction of both GC^+^ and GC^-^ TLS numbers in NeoTx patients was observed in the whole tissue and peritumoral region. In the intratumoral region, the difference was significant only for GC^+^ TLS. Strikingly, not a single tissue containing GC^+^ TLS was found in the NeoTx group, while seven samples in the PR group contained at least one GC^+^ TLS. To confirm this observation in an independent cohort, we took advantage of the multicenter PREOPANC phase III trial patient collective. In this study, resectable and borderline resectable PDAC patients were randomly assigned to PR (n=44) or NeoTx followed by resection (n=40) (48). Using routine H&E-stained sections, we assessed both peritumoral and intratumoral tissue regions for the number of GC^+^ and GC^-^ TLS and compared the number of TLS between treatment arms (**Figure 5B**). Also in this randomized cohort, patients who underwent PR displayed significantly higher TLS abundance compared to the NeoTx group. These differences were especially pronounced in the peritumoral region and highly significant for both GC^+^, GC^-^, and all TLS combined.

**Figure 6:**
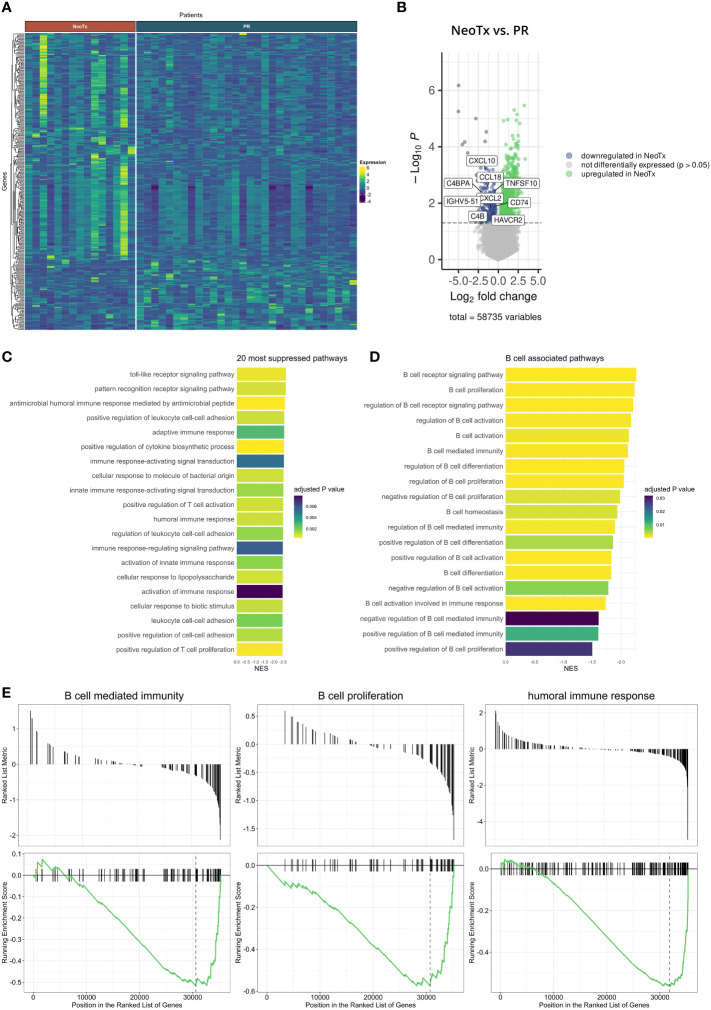
Bulk RNA sequencing analysis of NeoTx and PR PDAC patients. **(A)** Heatmap illustrating differentially expressed genes in PR (n=30) and NeoTx (n=15) patients. **(B)** Volcano plot displaying genes significantly up- or downregulated in NeoTx patients. **(C)** A bar chart visualizing the top 20 most suppressed pathways in NeoTx patients. **(D)** A bar chart displaying suppression of B cell-associated pathways in the NeoTx group. **(E)** Exemplary gene set enrichment plots for key B cell immunity pathways, including B cell-mediated immunity, B cell proliferation, and humoral immunity.

To further elucidate potential underlying mechanisms of the reduced TLS and B cell presence, we explored bulk RNAseq data in an additional cohort of 30 PR and 15 NeoTx patients (**Figure 6**). We identified 999 upregulated and 259 downregulated genes in the NeoTx group compared to PR patients (**Figures 6A, B**). Interestingly, the 20 most strongly suppressed pathways were dominated by immune-related pathways, including the humoral immune response (**Figure 6C**). This prompted us to further investigate B cell-associated pathways, which revealed that besides the B cell receptor signaling, also pathways of B cell proliferation, activation, and differentiation were negatively regulated in NeoTx patients (**Figure 6D**). **Figure 6E** displays exemplary geneset enrichment plots for B cell-mediated immunity, B cell proliferation, and the humoral immune response, illustrating the detrimental effect of NeoTx on the B cell compartment.

In summary, we observed a significantly reduced abundance of PDAC-infiltrating B cells, diminished presence of TLS, and suppressed immune-related and B cell-associated pathways in NeoTx patients.

### Plasma cells accumulate in CXCL12-rich areas around tumor-associated TLS in PDAC

**Figure 7:**
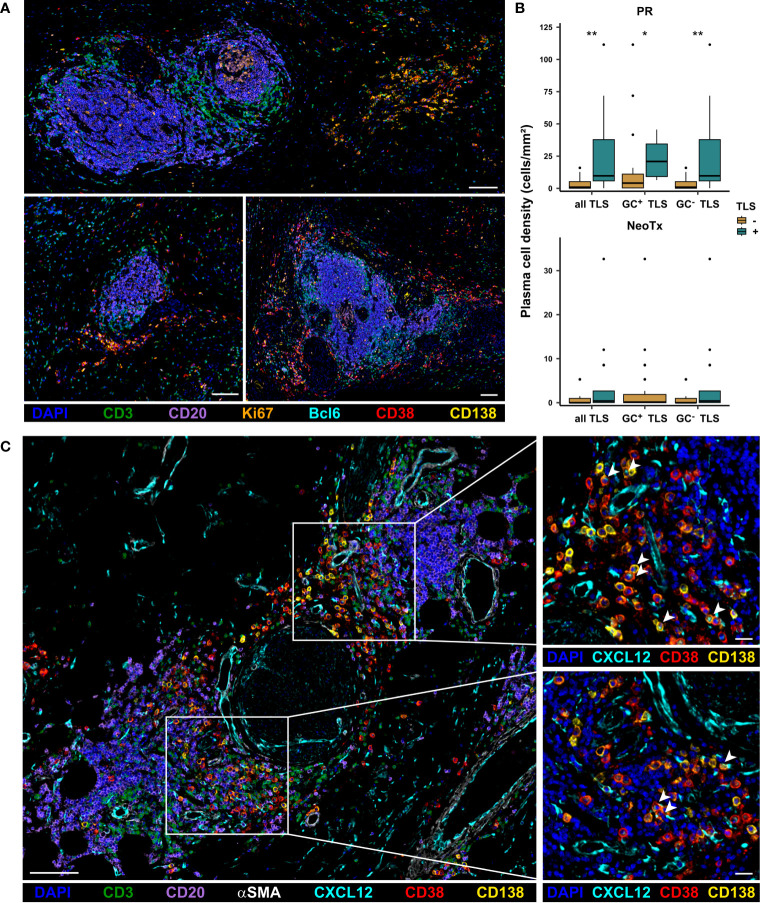
Association of TLS and CD38^+^CD138^+^ plasma cell presence in PDAC patients. **(A)** Representative images of plasma cell aggregates located in close proximity to TLS in PDAC patients. Tissues were stained for DAPI (blue), CD3 (green), CD20 (purple), Bcl6 (cyan), Ki67 (orange), CD38 (red), and CD138 (yellow). The scale bar is 100 µm. **(B)** Patients were stratified based on the presence of TLS and plasma cell frequencies in the stromal compartment of the intratumoral region were compared between TLS^+^ and TLS^-^ patients for both the PR and the NeoTx group. Significant differences were determined using the unpaired Wilcoxon test and are shown as * ≙ p-value ≤ 0.05 and ** ≙ p-value ≤ 0.01. **(C)** Representative image of PDAC tissues stained for DAPI (blue), CD3 (green), CD20 (purple), CXCL12 (cyan), αSMA (white), CD38 (red), and CD138 (yellow), including zoom-ins showing CXCL12 staining pattern in relation to plasma cell location. CXCL12^+^CD38^+^CD138^+^ plasma cells are marked by arrow-heads. The scale bar is 100 µm for the overview image (left) and 20 µm for the zoom-ins (right).

In contrast to pan B cells, GC B cells, and plasmablasts, which are mainly located within TLS, plasma cells were preferentially located in stromal regions outside TLS. It is known that TLS can harbor the differentiation of B cells into antibody-producing plasma cells, encouraging us to explore a potential link between TLS presence and plasma cell abundance. Interestingly, we observed that CD38^+^CD138^+^ plasma cells frequently formed aggregates, which are often located in close proximity to TLS (**Figure 7A**). We further found that plasma cells were significantly more frequent in TLS^+^ tissues than in TLS^-^ tissues in the PR group (**Figure 7B**). This held true for the overall presence of TLS, as well as the specific abundance of GC^+^ or GC^-^ TLS. While NeoTx patients lacked GC^+^ TLS, a similar trend was observed for GC^-^ TLS, although not reaching statistical significance. We thus hypothesized that the presence of TLS might favor the differentiation of B cells into Ig-producing plasma cells. Since plasma cells are known to migrate along CXCL12^+^ fibroblastic tracks (49), we conducted a co-staining of CD38, CD138, CD20, CD3, the fibroblast and pericyte marker alpha smooth muscle actin (αSMA), and CXCL12. TLS-associated CXCL12 displayed a perivascular and fibroblastic staining pattern, partly positive for αSMA (**Figure 7C**). Notably, aggregates of CD38^+^CD138^+^ plasma cells were often surrounded by prominent CXCL12 staining. In addition, CXCL12^+^CD38^+^CD138^+^ plasma cells were also observed, displaying a granular, endosomal CXCL12 staining pattern, potentially indicating an uptake of receptor-bound CXCL12.

### PDAC-infiltrating CD20^+^ B cells are associated with improved overall survival in PR patients

**Figure 8:**
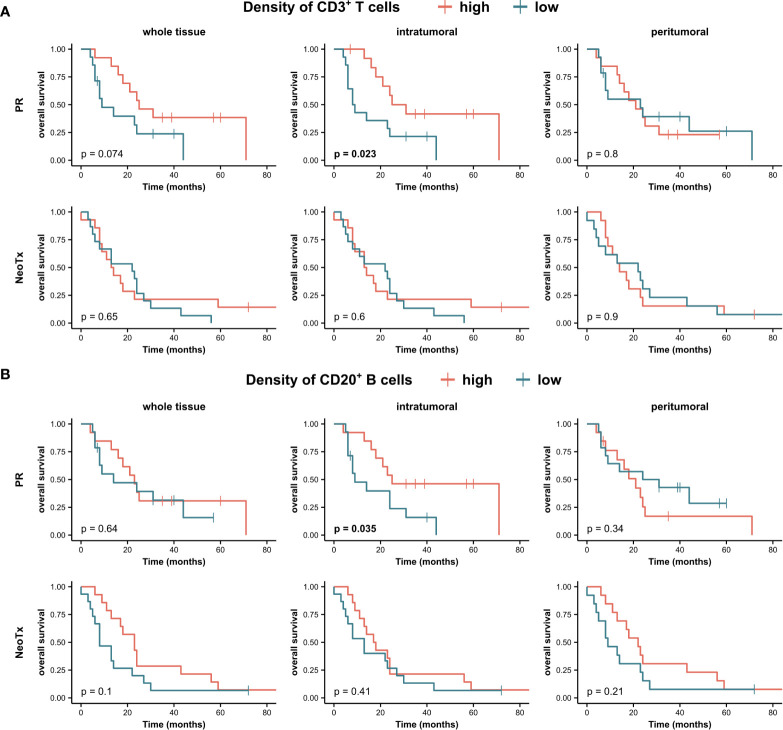
Association between tumor-infiltrating lymphocytes and overall survival (OS) of PDAC patients. Kaplan-Meier survival analysis of OS stratified by densities of **(A)** CD3^+^ T cell and **(B)** CD20^+^ B cells in the combined tissue class, all three tissue regions (whole tissue, intratumoral, peritumoral), and different treatment groups (PR, NeoTx). Patients were stratified by the median cell density. A log-rank test was performed, and p-values ≤ 0.05 were considered significant.

Subsequently, we examined a potential link between the frequency of PDAC-infiltrating lymphocytes and OS in both the PR and NeoTx groups (**Figure 8**). We first assessed the association of CD3^+^ T cell and CD20^+^ B cell frequencies in the combined compartment of all tissue regions. A higher frequency of intratumoral CD3^+^ T cells in the combined tissue compartment was associated with significantly longer OS in upfront resected patients (**Figure 8A**, p=0.023). While there was a similar trend in the whole tissue region of the PR group (p=0.074), no prognostic value of peritumoral CD3^+^ T cells was observed. In NeoTx patients, no association with survival was observed in any of the tissue regions. In addition, a higher frequency of CD20^+^ B cells in the intratumoral region was linked to significantly prolonged OS of the PR group (**Figure 8B**, p=0.035). Importantly, this finding remained true when focusing on non-TLS-associated B cells by analyzing the stromal compartment without TLS (**Supplementary Figure 4**, p=0.016). Interestingly, no prognostic significance of CD20^+^ B cells was found in the NeoTx group (**Figure 8B**).

### Proliferating CD20^+^Ki67^+^ B cells as an independent prognostic factor in NeoTx patients

**Figure 9:**
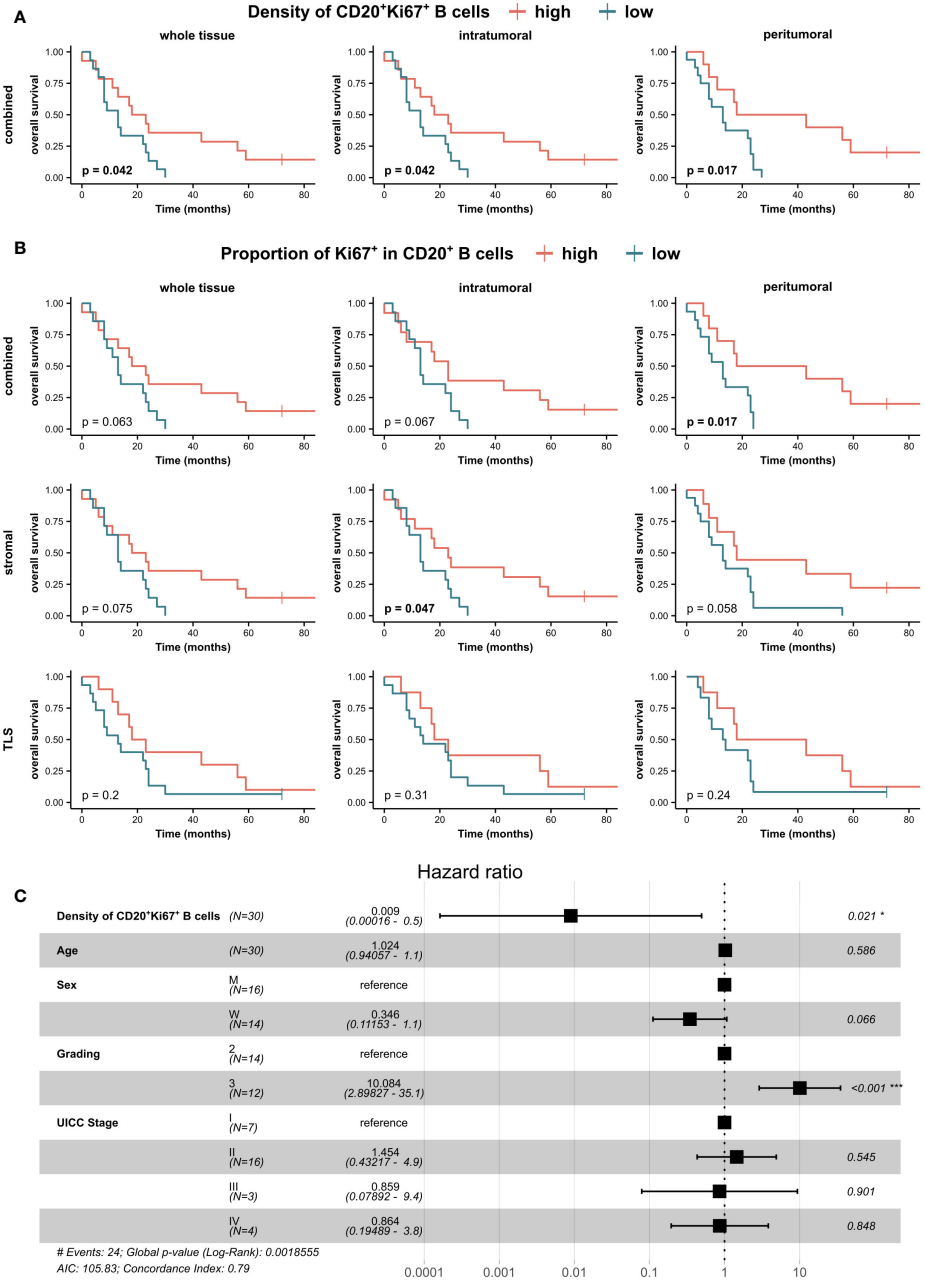
Clinical association of PDAC-infiltrating proliferating CD20^+^Ki67^+^ B cells in neoadjuvant-treated patients. Patients of the NeoTx group were stratified by the median of **(A)** CD20^+^Ki67^+^ B cell density in the combined tissue class and **(B)** Ki67^+^ proportion of CD20^+^ B cells in all three compartments (combined, stromal, TLS) and subjected to Kaplan-Meier survival analysis. A log-rank test was performed, and p-values ≤ 0.05 were considered significant. **(C)** Forest plot of Cox proportional hazard regression model for the risk of death including the density of CD20^+^Ki67^+^ B cells in the stromal compartment of the intratumoral tissue region, patient age, sex, grading, and UICC stage. Hazard ratios (HR) and 95% confidence intervals (CI) are shown. A log-rank test was performed, and p-values ≤ 0.05 were considered significant.

In addition, we investigated whether actively replicating immune cells, indicative of a restorative tumor immune microenvironment following NeoTx, were associated with OS (**Figure 9**). A higher frequency of CD20^+^Ki67^+^ B cells in the combined compartment was linked to significantly improved OS in all tumor regions of the NeoTx group (**Figure 9A**). Furthermore, a higher proportion of Ki67^+^ of all CD20^+^ B cells was significantly associated with longer OS in the combined tissue class of the peritumoral region (**Figure 9B**, p=0.017). Analyzing the stromal compartment and thus excluding TLS-associated B cells, this effect was significant in the intratumoral region (p=0.047) and displayed a strong trend in the peritumoral region (p=0.058). Interestingly, when focusing on the TLS compartment, the proportion of Ki67^+^ B cells held no prognostic value. To account for the effects of other covariates, we performed a multivariate Cox ph analysis (**Figure 9C**). With patient age, sex, tumor grading, and UICC stage as covariates, a higher frequency of intratumoral CD20^+^Ki67^+^ B cells in the stromal tissue compartment was significantly and independently correlated to a lower risk of death (HR=0.009, p=0.021).

## Discussion

The frequency and spatial distribution of tumor-infiltrating lymphocytes, as well as the presence of TLS, emerged as pivotal prognostic and predictive factors for the survival and treatment response of cancer patients (8, 9, 18, 25). Thus, uncovering the underlying mechanisms and their modulation by NeoTx regimens became increasingly relevant to harness existing antitumor immune responses. While the T cell compartment, both in the context of TLS and in general, is relatively well characterized in PDAC, studies investigating B cells are very limited and mainly relied on CD20 as a pan B cell marker so far (21, 23). Therefore, we investigated the effect of NeoTx on the frequency, spatial distribution, and clinical significance of different B cell subsets and TLS using a newly established 7-color mIHC panel and bulk RNAseq.

Since the *immunoscore* was established in colorectal cancer, the spatial distribution of tumor-infiltrating immune cells quickly emerged as a critical determinant of clinical outcomes in various cancers (50). Thus, we first assessed the densities of PDAC-infiltrating lymphocyte subsets in different tissue compartments (stromal, intraepithelial, TLS, and combined) and tissue regions (intratumoral, peritumoral, and whole tissue). Interestingly, we observed a higher T and B cell density in the peritumoral region compared to the intratumoral area. This is typically seen in immune-excluded tumors and was previously described for T cells and macrophages in PDAC (51–53). Various factors may impact the effective infiltration of lymphocytes, including physical barriers such as a dense stroma or functional barriers like gradients of chemo-repulsion (54). In a PDAC mouse model, Ly6C^low^ F4/80^+^ extratumoral macrophages were shown to restrict the infiltration of T cells into the tumor core and may thus function as gatekeepers (55). We further observed that the number of lymphocytes infiltrating the tumor epithelium was low, as most cells located in stromal regions and TLS. While the proportion of immune cells residing in the stroma is naturally higher, this effect seems especially pronounced in PDAC, as previously shown for T cells and macrophages (14, 56). This may be explained by the PDAC-characteristic dense, fibrotic desmoplastic stroma and extensive extracellular matrix, leading to lymphocytes sequestering within the stromal compartment (10).

Although B cells were described in PDAC before, to the best of our knowledge, a distinct and spatial analysis of B cell subsets, as well as their modulation by NeoTx, has not been reported so far. Using a novel mIHC panel, we identified proliferating B cells, GC B cells, plasmablasts, and plasma cells in PDAC tissues. As expected, both GC B cells and plasmablasts displayed extremely low average densities, detected only in about half of the cohort and preferentially located within TLS. In contrast, plasma cells were detected in most of the patients and resided within the stromal compartment. The analysis of TLS revealed that while many tissues harbored GC^-^ TLS, only 7 out of 58 patients presented with GC^+^ TLS, which matches the observation of very few GC B cell- and plasmablast-containing tissues. TLS, as specialized areas of B and T cell activation, are associated with improved survival in various cancer entities, including PDAC, and as shown by Gunderson et al., GC^+^ TLS are further associated with neoantigen burden and humoral immunity in PDAC (57). A recent study by Kinker et al. provided further evidence that a GC TLS signature is associated with improved survival in PDAC (58).

As NeoTx is the dominant treatment option for primarily non-resectable PDACs, several studies explored its effect on the immune contexture of PDAC, although with a strong focus on T cells and myeloid cells. We previously showed that although the total number of CD45^+^ cells per field of view was reduced, the NeoTx group displayed an increased proportion of CD8^+^ cells and decreased regulatory T cell proportions, suggesting an enrichment of antitumor immune cells following NeoTx (23). We further demonstrated that NeoTx patients displayed increased expression of proinflammatory cytokines produced by PDAC-infiltrating T cells (15). Regarding CD20^+^ B cells, absolute numbers, as well as the percentage of CD45^+^ cells, were reduced in the NeoTx group, although not significantly (23). Two studies relying on transcriptomic data further reported that B cell presence and function were reduced in neoadjuvant-treated patients (21, 22). Another recent study investigating TLS in PDAC described a lower frequency of CD20^+^ B cells in neoadjuvantly treated patients, but only in the TLS^+^ group (59). In our cohort, we observed that NeoTx patients displayed significantly lower numbers of pan B cells, proliferating B cells, GC B cells, plasmablasts, and plasma cells, while pan T cell frequencies were unaffected. Importantly, this effect persisted when focusing on B cells residing in the stroma outside TLS. Additionally, we demonstrated a significantly diminished proportion of proliferating B cells and GC B cells within the pan B cell population. This observation implies not only a quantitative alteration but also a shift in the functional properties of B cells after NeoTx. Furthermore, a striking reduction in the quantity of tumor-associated TLS was observed, which was especially pronounced for GC^+^ TLS, as not a single patient in the NeoTx group presented with GC^+^ TLS. We further validated these findings using the PREOPANC patient cohort as an independent study with randomized treatment arms. While the results were similar for both regions, the effect was more distinct in the peritumoral area. Our observations are further supported by Kuwabara et al., who reported a lower TLS area in neoadjuvant-treated patients, and two more studies that described a lower proportion of patients with TLS in the NeoTx group (59–61). In addition, Zou et al. showed that TLS in the NeoTx group were less abundant, smaller in size, and had lower maturation levels (59). A recent study by Kinker et al. revealed that tumor-reactive T cells exposed to TGF-β produce the B cell chemoattractant CXCL13, which may drive TLS formation (58). Utilizing bulk RNAseq data, we demonstrated that most of the 20 most suppressed pathways in NeoTx patients are immune response related, including the humoral immune response. Other B cell-associated pathways were also downregulated, further supporting our mIHC-based findings of B cell suppression in NeoTx patients. In addition, employing single cell RNAseq analysis may reveal altered gene expression profiles of B cells still present in tumors of NeoTx patients. While conventional anticancer treatments like chemotherapy harbor the potential to induce and improve tumor-specific immune responses, the cytotoxic properties also affect the present immune architecture. To further assess this effect, a matched cohort of pre-treatment biopsies and post-treatment resected tumors would be required, as results based on unmatched groups may be influenced by other factors. Additionally, immune cell subsets may differ in their susceptibility to DNA-damaging agents, as we found that T cells were not affected as strongly as B cells. Studies focusing on peripheral blood of chemotherapeutically treated cancer patients reported that B cells were the most dramatically affected immune cell subset, while T and natural killer cells were less susceptible (62–64). However, analyses of peripheral blood may not properly reflect intratumoral events. A PDAC study based on transcriptomic data also indicated that other immune cell subsets, such as CD8^+^ T cells, DCs, and macrophages, are not as strongly affected by chemotherapy as B cells or regulatory T cells (22).

One of the main antitumor properties of B cells is mediated by antibody-producing plasma cells, which have been associated with improved survival in multiple tumor entities, including triple-negative breast cancer, esophageal squamous cell carcinoma, ovarian, bladder, and gastric cancer (65–69). Interestingly, previous studies suggested that TLS can foster B cell differentiation into plasma cells (49, 66). In PDAC, several studies indicated the presence of plasma cells based on transcriptomic data and also reported a link between a plasma cell signature and improved survival (58, 70–72). In this study, we first reported the presence of PDAC-infiltrating CD38^+^CD138^+^ plasma cells, which were markedly reduced in neoadjuvant-treated patients and located mainly outside of TLS. We further discovered that in the PR group, patients with TLS displayed a significantly higher frequency of plasma cells and that clusters of plasma cells were often located in close proximity to TLS, suggesting that TLS can drive plasma cell differentiation in PDAC. Similar results were reported by Kroeger et al., who showed that ovarian cancer patients with TLS presented with higher plasma cell scores and that those plasma cells often resided in clusters close to tumor-associated TLS (66). A recent study in renal cell cancer uncovered that plasma cells disseminate from TLS along fibroblastic tracks containing CXCL12 (49). Furthermore, Ig-producing plasma cells were associated with IgG bound to tumor cells and an improved response to immunotherapy. By staining for CXCL12 and αSMA, we showed that plasma cell accumulations often colocalized with CXCL12^+^ fibroblasts near TLS, supporting the evidence of renal cell cancer. As suggested by Meylan et al., these CXCL12^+^ fibroblasts may resemble mesenchymal stromal cell-like fibroblasts in the bone marrow or follicular reticular cells, which promote the survival of both early and memory plasma cells (73–75). Interestingly, a significant proportion of plasma cells also displayed a distinct granular, endosomal staining pattern of CXCL12. Since plasma cells do not characteristically express CXCL12 themselves, this may be attributed to internalization of receptor-bound CXCL12. Upon binding to its cognate receptor CXCR4 and/or CXCR7 (ACKR3), the ligand-receptor complex is internalized and CXCL12 is delivered to lysosomes for degradation (76). This finding further supports our hypothesis of CXCL12-mediated plasma cell migration in proximity of TLS in PDAC.

Based on the drastic effects of NeoTx on the immune landscape of PDAC, we further explored the differential association of lymphocyte frequencies with OS in NeoTx and PR patients. So far, contradictory results regarding the prognostic value of CD20^+^ B cells in PDAC have been published, with several studies describing a positive impact on survival (20, 24, 77, 78), while one report observed a negative association (79). A meta-analysis integrating multiple studies reported no link between B cell frequencies and survival in PDAC (80). In our study, high intratumoral CD3^+^ T cell and CD20^+^ B cell frequencies were linked to significantly longer OS in the PR cohort but harbored no beneficial effects in the NeoTx group. This suggests that not only the number of infiltrating lymphocytes is reduced, but that also their functional properties are impaired upon NeoTx. Additionally, spatial analyses revealed that extra-TLS B cells also provided a survival benefit in the PR group. This contradicts findings from Castino et al., who reported that B cells were only associated with improved survival of PDAC patients when organized in TLS (24). However, their cohort of 104 patients included both NeoTx and PR patients without a subset analysis of treatment groups. Another study described a positive effect of B cell aggregates on the survival of primary resected PDAC patients but only in the tumor infiltration zone (20). In contrast, we did not observe any link between peritumoral B cell frequencies and survival in either of the treatment groups.

When exploring potentially prognostic B cell subsets in the NeoTx cohort, we found that increased frequencies of proliferating CD20^+^Ki67^+^ B cells were significantly associated with longer OS. Furthermore, the proportion of these cells in the total B cell population correlated with improved OS in the stromal compartment of the intratumoral region and the combined tissue category in the peritumoral region. Of note, these findings lacked statistical significance when analyzing only the TLS compartment, suggesting a TLS-independent mechanism. A multivariate Cox ph analysis including patient age, sex, tumor grading, and UICC stage as covariates validated the density of CD20^+^Ki67^+^ B cells as an independent prognostic factor for a reduced risk of death in the NeoTx cohort. While proliferating CD8^+^Ki67^+^ T cells have been associated with improved survival in various tumor entities, less is known about the role of proliferating intratumoral B cells (81). Mostly, CD20^+^Ki67^+^ B cells are used to identify mature TLS and are not analyzed independently of TLS. However, one glioblastoma study reported that CD20^+^Ki67^+^ B cells were linked to improved OS (82). Interestingly, we observed this correlation only in the NeoTx group, which exhibited significantly lower B cell frequencies compared to PR patients. The presence of actively replicating B cells might reflect a restorative post-therapy immune microenvironment with a flourishing adaptive antitumor immune response.

In conclusion, we found that neoadjuvant-treated patients displayed significantly lower numbers of all B cell subsets in comparison to the PR group, suggesting a detrimental effect of NeoTx on the B cell compartment. Importantly, not only the frequency but also the functional composition was altered, displayed by lower proportions of proliferating B cells and GC B cells. While this was accompanied by a drastically lower number of TLS in the NeoTx group, non-TLS-associated B cells were also affected. Additionally, transcriptomic analyses validated significantly suppressed B cell-associated pathways in the NeoTx group. We further showed that PDAC-infiltrating plasma cells were significantly more frequent in TLS^+^ tissues. These plasma cells are often aggregated in clusters, found in the vicinity of TLS and accompanied by CXCL12^+^ fibroblastic tracks, suggesting that TLS can foster plasma cell differentiation in PDAC. Lastly, increased frequencies of PDAC-infiltrating B cells were associated with improved OS only in the PR group, suggesting also a loss of functional properties in the NeoTx group. However, proliferating CD20^+^Ki67^+^ B cells emerged as an independent prognostic factor in neoadjuvant-treated patients, possibly reflecting a re-induction of B cell immunity after therapy. While these findings revealed a markedly suppressed B cell-mediated immune response in NeoTx patients, accurately matched pre- and post-therapy paired samples are necessary to track the effect of NeoTx on different B cell subsets in a longitudinal manner. Additionally, a more detailed analysis is required to understand the molecular mechanisms of TLS-induced plasma cell differentiation and their role in antitumor immunity. For example, BCR and Ig sequencing may be leveraged to reveal whether TLS-associated plasma cell-derived IgG is relevant for tumor cell apoptosis and linked to clinical outcomes. In addition, further studies investigating potential effects on the efficacy of immunotherapies are required. An improved understanding of the immunomodulatory effects of NeoTx is crucial to overcoming the immunosuppressive pancreatic TME, selecting patients for the most effective treatment modalities, and improving the devastatingly low survival of PDAC patients.

## Data availability statement

The original contributions presented in the study are included in the article/**Supplementary Material**. Further inquiries can be directed to the corresponding authors.

## Ethics statement

This study was approved by the Ethics Committee of the Technical University of Munich. The studies were conducted in accordance with the local legislation and institutional requirements. The participants provided their written informed consent to participate in this study.

## Author contributions

LRu: Conceptualization, Data curation, Investigation, Methodology, Supervision, Visualization, Writing – original draft, Writing – review & editing. ID: Conceptualization, Data curation, Investigation, Methodology, Writing – original draft, Writing – review & editing. MK: Conceptualization, Data curation, Investigation, Methodology, Visualization, Writing – original draft, Writing – review & editing. US: Supervision, Writing – original draft, Writing – review & editing. AM: Resources, Writing – original draft, Writing – review & editing. KS: Resources, Writing – original draft, Writing – review & editing. CWFE: Resources, Writing – original draft, Writing – review & editing. LRi: Methodology, Writing – original draft, Writing – review & editing. RI: Methodology, Writing – original draft, Writing – review & editing. CJ: Resources, Writing – original draft, Writing – review & editing. HF: Resources, Writing – original draft, Writing – review & editing. CHJE: Resources, Writing – original draft, Writing – review & editing. IED: Funding acquisition, Supervision, Writing – original draft, Writing – review & editing. CM: Conceptualization, Data curation, Funding acquisition, Investigation, Writing – original draft, Writing – review & editing. MS: Conceptualization, Funding acquisition, Writing – original draft, Writing – review & editing.
