# Supplementary material for: Neoadjuvant chemotherapy is associated with suppression of the B cell-centered immune landscape in pancreatic ductal adenocarcinoma
Source: Front Immunol. 2024 Apr 2;15:1378190. doi: 10.3389/fimmu.2024.1378190 (PMC11018975; doi:10.3389/fimmu.2024.1378190)
Supplement: Supplementary file 2 [file DataSheet_2.docx]

Supplementary Material

**Supplementary Table 1.** Clinicopathological characteristics of PDAC patients in the bulk RNAseq cohort. For continuous variables, the median is displayed with the range in brackets. For categorical variables, the number of patients with the percentage of all patients in brackets is shown.

|  | **PR (n=30)** | **NeoTx (n=15)** |
| --- | --- | --- |
| **Sex** Male Female | 15 (50%) 15 (50%) | 6 (40%) 9 (60%) |
| **Age** (years) | 70 (47-87) | 62 (43-79) |
| **Tumor size** (mm) | 32 (18-80) | 39 (20-70) |
| **T Status** T1-2 T3-4 | 18 (60%) 12 (40%) | 7 (46.7%) 8 (53.3%) |
| **N Status** N0 N1 N2 | 11 (36,7%) 6 (20%) 13 (43.3%) | 5 (33.3%) 5 (33.3%) 5 (33.3%) |
| **M Status** M0 M1 | 25 (83.3%) 5 (16.7%) | 14 (93.3%) 1 (6.7%) |
| **Grading** G1 G2 G3 | 1 (3.3%) 18 (60%) 11 (36.7%) | 1 (10%) 7 (70%) 2 (20%) |
| **Resection Status** R0 R1 | 9 (33.3%) 18 (66.7%) | 1 (8.3%) 11 (91.7%) |
| **Tumor Localisation** Head Body Tail | 19 (63.3%) 7 (23.3%) 4 (13.3%) | 9 (60%) 2 (13.3%) 4 (26.7%) |

**Supplementary Table 2.** Neoadjuvant therapy regimen of PDAC patients included in the bulk RNAseq cohort.

|  | CTx only (n=13) | Additional RTx (n=2) |
| --- | --- | --- |
| Gemcitabine | 4 (30.8%) | 0 (0%) |
| FOLFIRINOX | 6 (46.1%) | 0 (0%) |
| Others | 3 (23.1%) | 1 (50%) |
| No information | 0 (0%) | 1 (50%) |

**Supplementary Table 3.** Clinicopathological characteristics of PDAC patients included in the PREOPANC trial for the manual counting of TLS in H&E-stained sections. For continuous variables, the median is displayed with the range in brackets. For categorical variables, the number of patients with the percentage of all patients in brackets is shown.

|  | **PR (n=44)** | **NeoTx (n=40)** |
| --- | --- | --- |
| **Sex** Male Female | 29 (65.9%) 15 (34.1%) | 21 (52.5%) 19 (47.5%) |
| **Age** (years) | 67 (40-80) | 66 (42-80) |
| **Tumor size** (mm) | 30 (19-50) | 32 (15-64) |
| **T Status** T1-2 T3-4 | 1 (2.3%) 43 (97.7%) | 10 (25%) 30 (75%) |
| **N Status** N0 N1 | 11 (25%) 33 (75%) | 30 (75%) 10 (25%) |
| **M Status** M0 M1 | 44 (100%) 0 (0%) | 40 (100%) 0 (0%) |
| **Grading** G1 G2 G3 | 2 (5.6%) 26 (72.2%) 8 (22.2%) | 4 (12.1%) 17 (51.5%) 12 (36.4%) |
| **Resection Status** R0 R1 | 22 (50%) 22 (50%) | 29 (72.5%) 11 (27.5%) |
| **Tumor Localisation** Head Body Tail | 42 (95.5%) 2 (4.5%) 0 (0%) | 34 (85%) 4 (10%) 2 (5%) |

**Supplementary Table 4.** Antibodies, Opal fluorophores, and dilutions used for multiplex immunohistochemistry.

| **Antibody** | **Dilution** | **Supplier** | **Incubation** | **Clone** | **Opal** | **Dilution** |
| --- | --- | --- | --- | --- | --- | --- |
| Anti-αSMA | 1:100 | Cell Signaling Technology, Inc. | 32 min,  RT | D4K9N | 690 | 1:100 |
| Anti-Bcl6 | 1:200 | Abcam plc. | 16 min,  RT | EPR11410-43 | 620 | 1:200 |
| Anti-CD138 | 1:75 | Cell Signaling Technology, Inc. | 32 min,  36 °C | IHC138 | 540 | 1:200 |
| Anti-CD20 | 1:400 | ThermoFisher Scientific | 16 min,  RT | L26 | 650 | 1:700 |
| Anti-CD3 | prediluted | Ventana Medical Systems, Inc. | 32 min,  RT | 2GV6 | 520 | 1:75 |
| Anti-CD38 | 1:900 | Cell Signaling Technology, Inc. | 32 min,  36 °C | E7Z8C | 570 | 1:1000 |
| Anti-CXCL12 | 1:75 | Cell Signaling Technology, Inc. | 32 min,  36 °C | D8G6H | 620 | 1:75 |
| Anti-Ki67 | 1:75 | Agilent Technologies, Inc. | 32 min,  36 °C | MIB-1 | 690 | 1:75 |


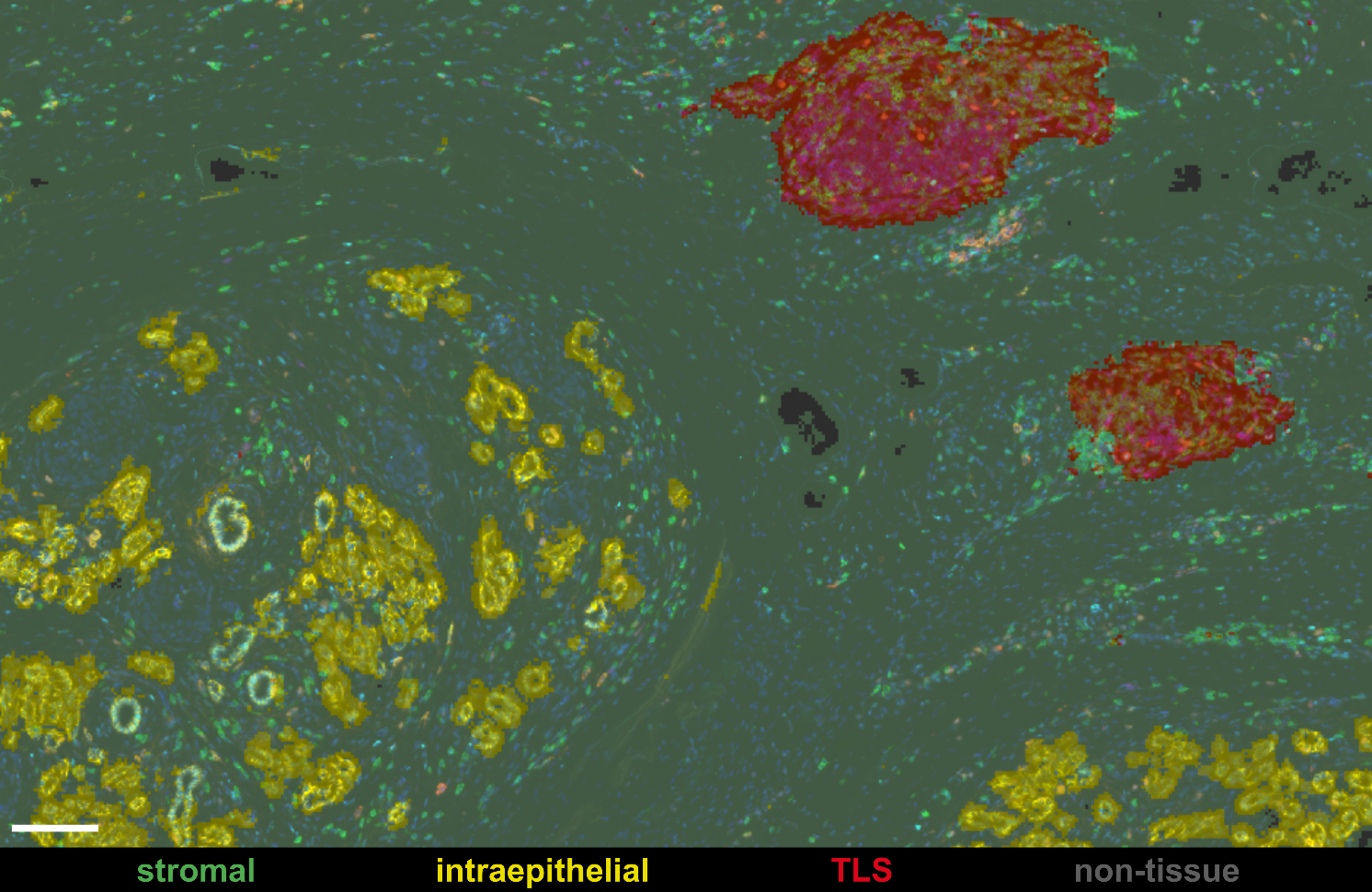


**Supplementary Figure 1: Representative image of the trained pixel classifier**. All tissue compartments (stromal in green, intraepithelial in yellow, TLS in red, non-tissue in gray) were trained on a training sparse image of 100 representative MSIs and validated on a separate set of 100 MSIs. The scale bar is 100 µm.


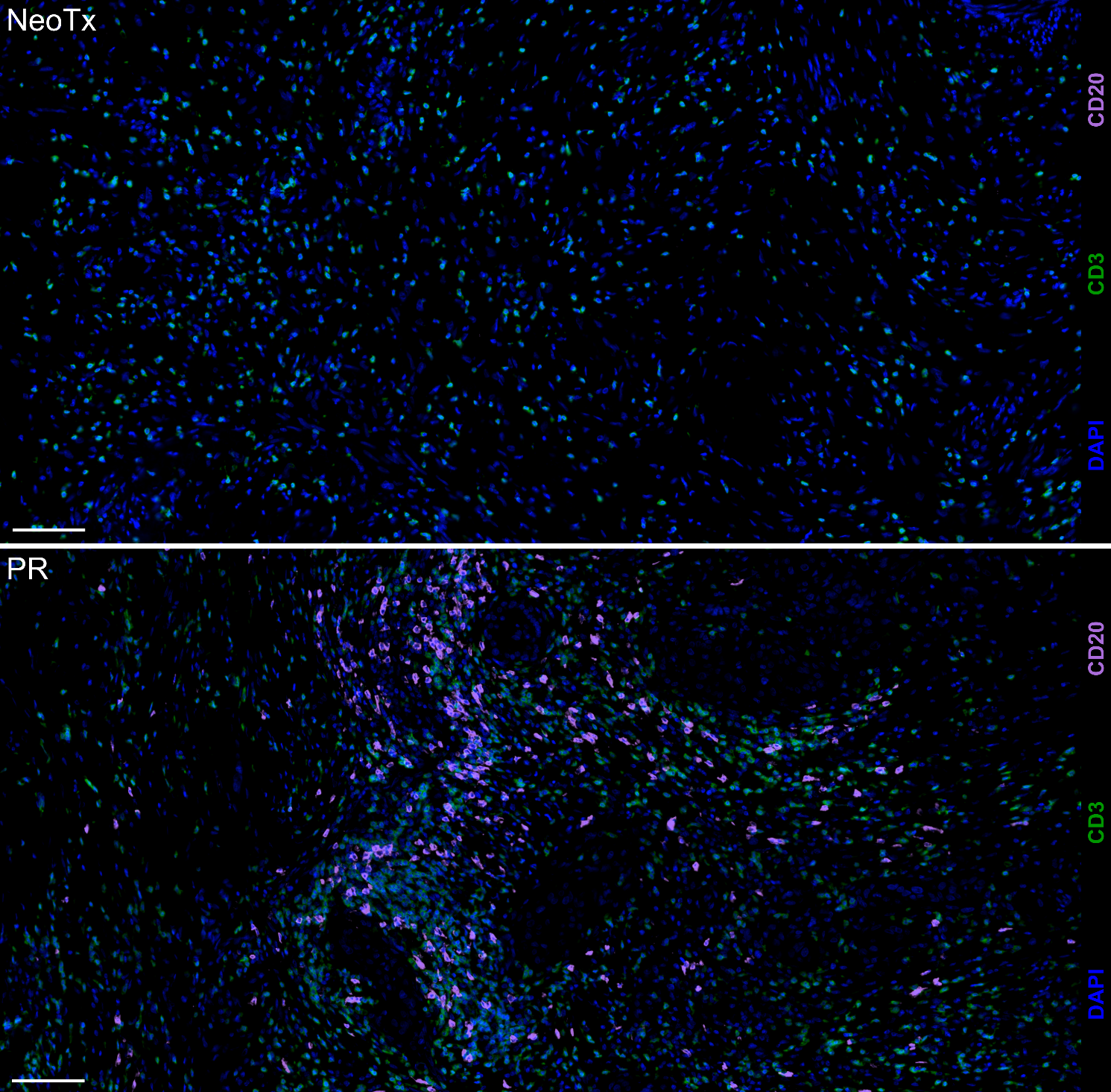


**Supplementary Figure 2: Multicolor images of a NeoTx and a PR patient.** Representative images of a NeoTx patient with low B cell density and a PR patient with high B cell infiltration stained for DAPI (blue), CD3 (green), and CD20 (purple). The scale bar is 100 µm.


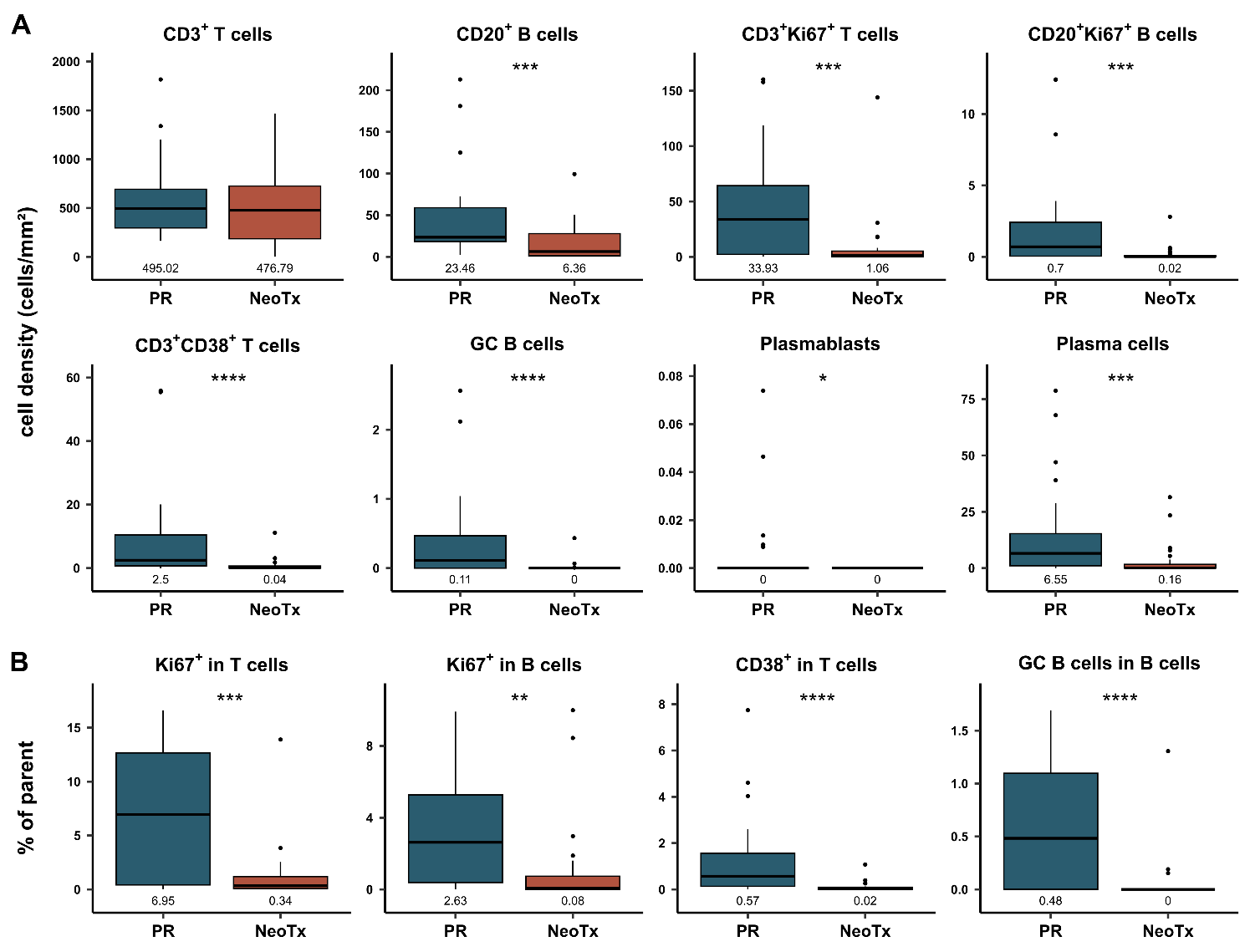


**Supplementary Figure 3: Comparative analysis of T and B cell frequencies and composition in the stromal tissue compartment of primarily resected (PR) and neoadjuvantly treated (NeoTx) patients.** (A) Cell densities (cells/mm²) in the stromal compartment of the whole tissue region were assessed for the main cell types and compared between the PR (n=28) and NeoTx (n=30) group. (B) Proportion of marker-positive cells in the stromal compartment of whole tissue region were compared between treatment groups. Median values are displayed, and significant differences were determined using the unpaired Wilcoxon test and are shown as * ≙ p-value ≤ 0.05, ** ≙ p-value ≤ 0.01, *** ≙ p-value ≤ 0.001, and **** ≙ p-value ≤ 0.0001.


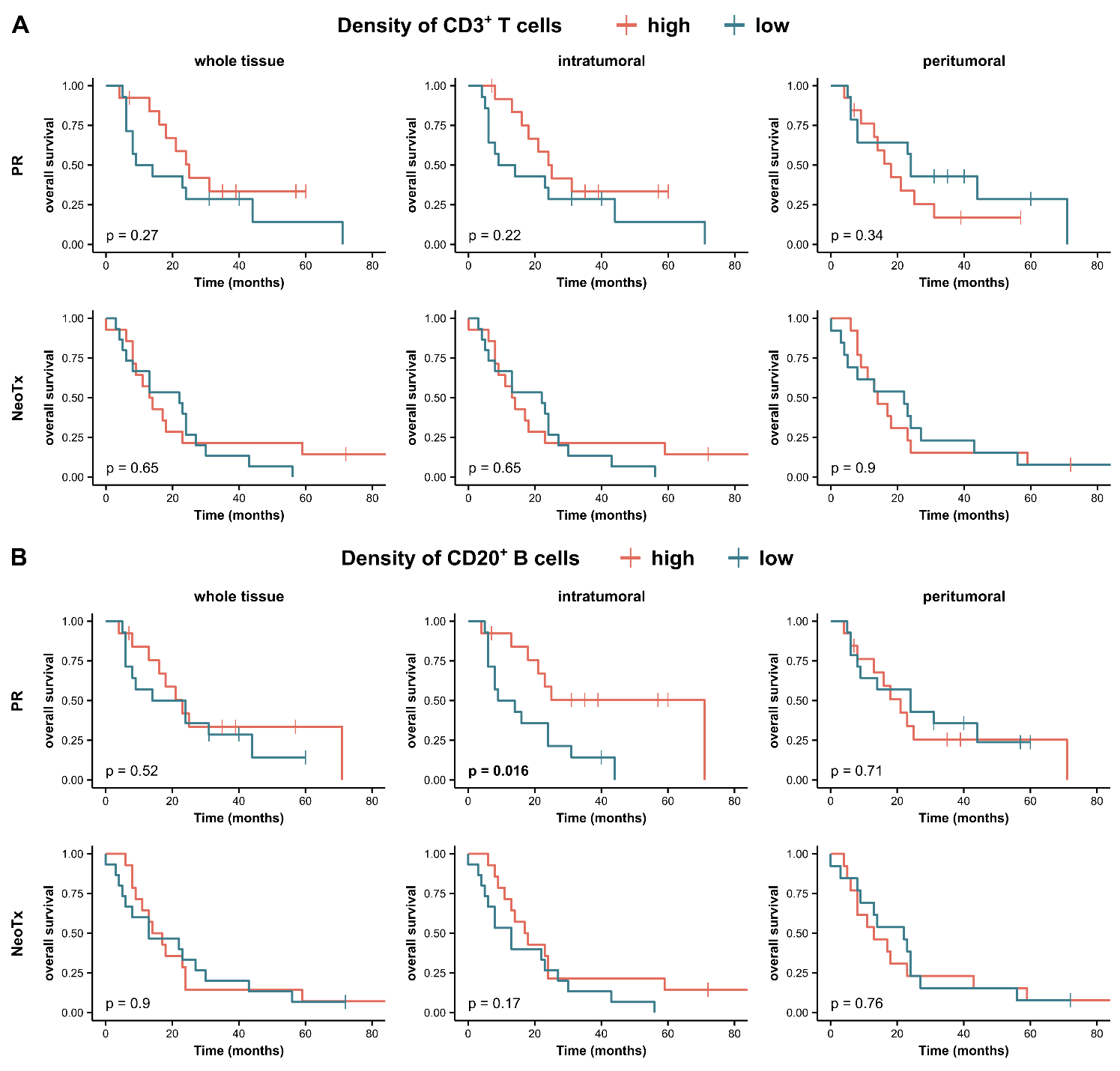


**Supplementary Figure 4: Association between tumor-infiltrating lymphocytes and overall survival (OS) of PDAC patients**. Kaplan-Meier survival analysis of OS stratified by densities of (A) CD3^+^ T cell and (B) CD20^+^ B cells in the stromal tissue compartment, all three tissue regions (whole tissue, intratumoral, peritumoral), and different treatment groups (PR, NeoTx). Patients were stratified by the median cell density. Log-rank test was performed and p-values ≤ 0.05 were considered significant.
